# COPD online-rehabilitation versus conventional COPD rehabilitation – rationale and design for a multicenter randomized controlled trial study protocol (CORe trial)

**DOI:** 10.1186/s12890-017-0488-1

**Published:** 2017-11-16

**Authors:** Henrik Hansen, Theresa Bieler, Nina Beyer, Nina Godtfredsen, Thomas Kallemose, Anne Frølich

**Affiliations:** 1 Research Unit for Chronic Diseases and Telemedicine, Bispebjerg and Frederiksberg Hospital, University of Copenhagen, Bispebjerg Bakke 23, 2450 Copenhagen, NV Denmark; 2 Department of Physical & Occupational Therapy, Bispebjerg and Frederiksberg Hospital, University of Copenhagen, Copenhagen, Denmark; 3 Institute for Clinical Medicine, University of Copenhagen, Copenhagen, Denmark; 4 Department of Respiratory Medicine, Hvidovre University Hospital, Hvidovre, Denmark; 5 Clinical Research Center, Hvidovre University Hospital, Hvidovre, Denmark; 6 Research Unit for Chronic Diseases and Telemedicine, University Hospital Bispebjerg and Frederiksberg, Bispebjerg Bakke 23, 2450 Copenhagen, NV Denmark

**Keywords:** COPD, Tele-rehabilitation, Pulmonary rehabilitation, Randomized controlled trial, Multicenter, Exercise, Quality of life

## Abstract

**Background:**

Rehabilitation of patients with chronic obstructive pulmonary disease (COPD) is a key treatment in COPD. However, despite the existing evidence and a strong recommendation from lung associations worldwide, 50% of patients with COPD decline to participate in COPD rehabilitation program and 30–50% drop-out before completion. The main reasons are severe symptoms, inflexible accessibility and necessity for transportation. Currently there are no well-established and evident rehabilitation alternatives. Supervised online screen rehabilitation could be a useful approach to increase accessibility and compliance. The aim of this multicenter RCT study is to compare the potential benefits of a 10-week online COPD rehabilitation program (CORe) with conventional outpatient COPD rehabilitation (CCRe).

**Methods:**

This study is a randomized assessor- and statistician blinded superiority multicenter trial with two parallel groups, employing 1:1 allocation to the intervention and the comparison group.On the basis of a sample size calculation, 134 patients with severe or very severe COPD and eligible to conventional hospital based outpatient COPD rehabilitation will be included and randomized from eight different hospitals. The CORe intervention group receives group supervised resistance- and endurance training and patient education, 60 min, three times/week for 10 weeks at home via online-screen. The CCRe comparison group receives group based supervised resistance- and endurance training and patient education, 90 min, two times/week for 10 weeks (two hospitals) or 12 weeks (six hospitals) in groups at the local hospital. The primary outcome is change in the 6-min walking distance after 10/12 weeks; the secondary outcomes are changes in 30 s sit-to-stand chair test, physical activity level, symptoms, anxiety and depression symptoms, disease specific and generic quality of life. Primary endpoint is 10/12 weeks from baseline, while secondary endpoints are 22, 36, 62 weeks from baseline assessments.

**Discussion:**

The study will likely contribute to knowledge regarding COPD tele-rehabilitation and to which extent it is more feasible and thereby more efficient than conventional COPD rehabilitation in patients with severe and very severe COPD.

**Trial registration:**

Clinicaltrials.gov Identifier: NCT02667171. Registration data: January 28th 2016.

## Background

Chronic Obstructive Pulmonary Disease (COPD) is a major cause of chronic morbidity and the fourth leading cause of death worldwide [1] (GOLD). COPD is characterized by increasing respiratory symptoms, frequent exacerbations and disability in activities of daily living. International and national guidelines emphasize that COPD rehabilitation is a key cornerstone in the standard treatment of COPD together with smoking cessation and pharmacological treatment [2–6]. The core elements of COPD rehabilitation is physical exercise training, patient-directed education and smoking cessation support, which are recommended as mandatory in standard COPD rehabilitation programs [2–6]. It is evident that COPD rehabilitation result in moderate to large clinically relevant improvements in quality of life, symptoms, anxiety and depression, walking distance, exercise tolerance and physical function in patients with mild as well as very severe stable COPD and in patients with acute phases of exacerbation [2–7]. However, approximately 50% of the patients with severe and very severe COPD decline to participate in COPD rehabilitation programs and 30–50% drop out before completion of the program [8–12]. The main reasons are severe symptoms and exacerbations, transportation distance and lack of energy [8–12]. There are no well-established rehabilitation alternatives for these patients, but supervised COPD tele-rehabilitation in groups delivered by health professionals in the patients’ own home via a computer, tablet or television screen could be a useful approach in terms of increasing compliance and adherence. Most studies on tele-rehabilitation in COPD have been non-randomized descriptive feasibility studies and they have reported promising effects on symptoms, physical function and quality of life [13–18]. Recently, results from three randomized controlled trials on tele-rehabilitation have been published [19–21]. In one study by Chaplin et al. (*n* = 103) [20] per protocol analyses showed equal results in regard to shuttle walk performance and COPD related symptoms when comparing effects of a short term (6–8 weeks) unsupervised web-based individually tailored home exercise program with a 7 week conventional pulmonary rehabilitation program comprising exercise and education. However, the dropout rate was higher in the web-based program (57% vs 23%). Another study by Vasilopoulou et al. (*n* = 150)^21^ showed that compared to a control group (usual care) the risk of acute COPD exacerbation and hospitalization was similar following a 12 months unsupervised individually tailored maintenance tele-rehabilitation exercise program combined with a weekly multidisciplinary telephone counseling and a 12 months supervised pulmonary maintenance rehabilitation program twice weekly at the hospital. However, compared to the other groups the tele-rehabilitation group had a reduced risk for visits to the emergency department. Finally, a small study by Tsai et al. (*n* = 37) [19] compared results from a non-exercising control group with results from a supervised 8-week tele-rehabilitation program in groups of 2–4 patients who could see and talk to both the physiotherapist and the other participants. The study showed significantly greater improvements in the endurance shuttle walk time (but not in the incremental shuttle walk time or the 6MWT), symptoms of anxiety and depression, and self-efficacy following the supervised tele-rehabilitation program.

Thus, the new results are promising, additional effects from face-to-face supervised COPD tele-rehabilitation in groups compared to outpatient supervised COPD rehabilitation in groups’ remains unanswered, i.e. potential higher compliance and effects when supervised and potentially better resource utilization when delivered in groups. To our knowledge this study protocol currently describes the first RCT study planned to investigate effects from equally face-to-face supervised and group based COPD rehabilitation in outpatient and tele-rehabilitation setting [22].

The purpose of our RCT study is to investigate the potential benefits of a 10-week supervised online COPD rehabilitation program (CORe) with conventional supervised COPD rehabilitation (CCRe) on walking distance (primary outcome), muscle endurance, physical activity level, quality of life, and COPD symptoms after completion of the intervention and at follow-up 3, 6 and 12 month later in patients with severe and very severe (stage III-IV) COPD. This paper describes the rationale and design of the study.

## Methods

### Study principles

**Fig. 1:**
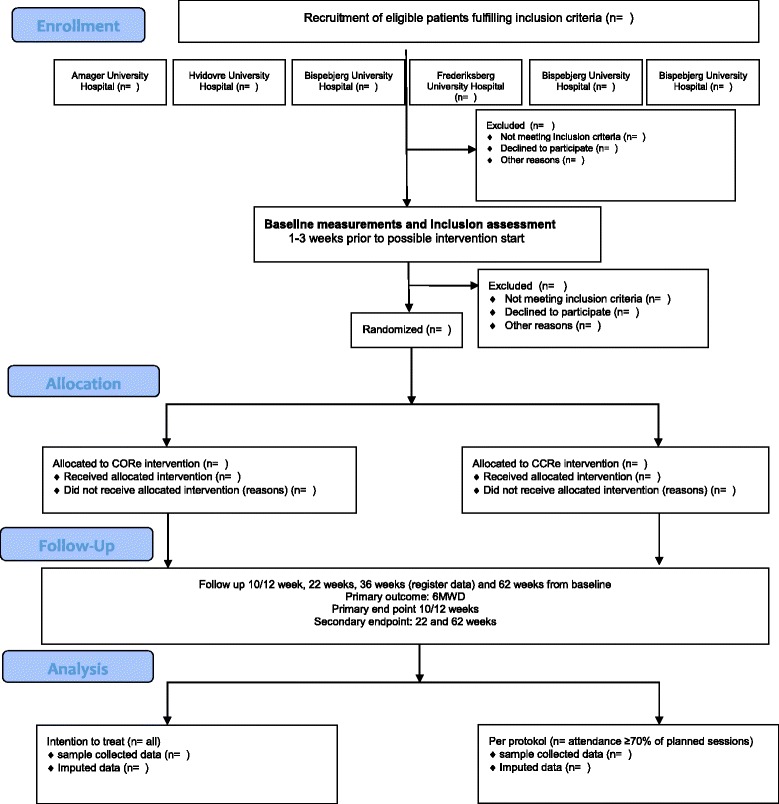
Consolidate standards of reporting trials (CONSORT) flow diagram of trial design

The protocol follows the SPIRIT 2013 (Standard Protocol Items: Recommendations for interventional Trials) and the Template for Interventions Description and Replication (TIDieR) checklist for description of the interventions [23, 24]. Once completed the reporting will follow the CONSORT (Consolidated Standards of Reporting Trials) Statement for non-pharmacologic trials [25] (Fig. 1).

### Study design

This study is a randomized controlled, assessor- and statistician blinded, superiority, multicentre trial with two parallel-groups. The trial investigates the effect of supervised COPD Online rehabilitation in groups, delivered by health professionals in the patients’ own home via a computer, in patients with severe and very severe (stage III-IV) COPD (ClinicalTrial.gov-identifier: NCT02667171). Patients from the University hospitals in the Capital Region of Denmark will be randomized to the supervised group-based online COPD rehabilitation (CORe) or to the conventional supervised COPD rehabilitation program (CCRe). The primary outcome will be the 6-min walking distance after completion of the COPD rehabilitation program (primary end point at 10/12 weeks). In addition, the study collects follow-up data at 3, 6 and 12 months after completion of the program which will be published in a separate paper (Fig. 1 and Table 1).

**Table 1:** Study measures and outcomes to be collected

Variable	Baseline	10/12 weeks (post)	3-month follow-up (22-weeks)	6-month follow-up (36-weeks)	12-month follow-up (62-weeks)
Primary outcomes					
6 min walk distance (6MWD)	X	X	X		X
Secondary outcomes					
30s sit-to-stand test (30STS)	X	X	X		X
Clinical COPD Questionnaire (CCQ)	X	X	X		X
COPD Assessment Test (CAT)	X	X	X		X
Hospital Anxiety Depression Scale	X	X	X		X
EuroQol 5D (3-L)	X	X	X		X
Other variables and outcomes					
Attendance in rehabilitation		X			
Number of COPD related hospital admissions	X	X	X	X	X
Number of COPD hospital days	X	X	X	X	X
COPD related outpatient visits	X	X	X	X	X
Number of COPD exacerbations	X	X	X	X	X
Mortality	X	X	X	X	X
Exploratory outcome					
24 h–mobility (ActivePAL3tm; 5 days)	X	X	X		X
Descriptive variables					
Lung function	X				X
FVC	X				X
FEV1	X				X
FEV1/FVC%	X				X
FEV1% expected	X				X
Charlson morbity Index	X				X
Anthropometric measures					
Gender	X				
Age	X				
Weight	X	X	X		X
Height	X	X	X		X
Body Mass Index (BMI)	X	X	X		X
Self-reported measures					
Smoking status	X	X	X		X
Pharmacologic treatment	X	X	X		X

The primary hypothesis is that CORe is superior to CCRe due to a higher compliance and adherence to the CORe program. We expect a mean between-group difference of 26 m in the 6-min walk test after completion of the COPD rehabilitation programs (primary endpoint at 12-weeks).

We also expect clinically relevant improvements in both groups as a results of completing the COPD rehabilitation (Table 1).

#### Study setting and study population

The trial is conducted at the Respiratory and Physiotherapy Departments of eight hospitals in the capital region of Denmark. The participating hospitals are Amager, Hvidovre, Bispebjerg, Frederiksberg, Herlev, Gentofte, Frederikssund and Hillerød University Hospitals, University of Copenhagen. Recruitment of patients with severe and very severe COPD and collection of data started March the 18th, 2016 and is scheduled to continue until December 31st 2017 (clinicaltrial.gov registration on January 12th, 2016). The participating hospitals will provide monthly reports on patients who accept or decline to participate and reasons for this. The recruitment will be facilitated by a steering committee with members from the departments of the participating hospitals. The investigator (HH) provides quarterly updates on the recruitment progress and participates in meetings with the clinical staff when requested.

#### Eligibility criteria

Potentially eligible patients will be identified and recruited by respiratory nurses during out-patient COPD control visits. The nurses determine eligibility according to the inclusion and exclusion criteria listed below:

#### Inclusion criteria

1. Age 18 years or older
2. Clinical diagnosis of COPD defined as the ratio of forced expiratory volume at 1 s (FEV1) to forced vital capacity (FVC) < 0.70 and no history of asthma
3. FEV1 < 50%, corresponding to severe or very severe airflow limitation
4. Symptoms equivalent to the Medical Research Council dyspnea scale (MRC) from 2 to 5

#### Exclusion criteria

1. Participation in/or recent completion of pulmonary rehabilitation within the last 6 months before start of intervention
2. Dementia/ Cognitive impairment or symptomatic psychiatric illness
3. An impaired hearing and / or vision disability which means that the instructions are not understood
4. Unable to understand and speak Danish
5. Unable to read Danish
6. Severe co-morbidity which means that exercise is contraindicated

Eligible patients receive written information of the study by the respiratory nurses and verbal information about the study is given by the investigator or project staff. The investigator ensures that all questions regarding participation are answered before the patient is asked to participate in the study. According to the ethical guidelines for medical research in Denmark, all patients are encouraged to consider consent for at least 24-h before making the decision. Patients who agree to participate will be asked to sign an informed consent form to be included in the study. The patient will keep the original document and a copy will be archived with the Case Report Form (CRF).

### Randomization

Following baseline assessments patients will be randomly allocated to the intervention group (CORe) or the comparison group (CCRe). Randomization will follow a computer-generated block randomization list, block size 2, made by a biostatistician (TK). The randomization will be a 1:1 randomization block from each recruiting hospitals.

### Blinding

To ensure concealment of allocation a senior manager from another research department with no interest in the project, will provide the draw and will be responsible for the randomization list, which will not be available to the investigator. The senior manager will inform the investigator about the allocation, and the investigator or the project staff will subsequently inform the patient about the allocation and when to begin CORe or CCRe.

All assessors are blinded to group allocation and previous test results. Due to the nature of the study the patients cannot be blinded, but prior to the assessments they are reminded not to disclose their group allocation to the assessors. In case of failure keeping the outcome assessor blinded (that is, if a participant reveals his/her allocation) a second assessor will be available to step in and conduct the assessment on another day.

To avoid experimenter’s (subconscious) bias the biostatistician who perform the data analyses and validate the results will be blinded to group allocation. The research group will interpret the results, and the conclusion will be prepared in two versions before the allocation code is broken (one assuming that arm A is the intervention, and one assuming that arm B is the intervention).

### Sample size

For the study’s primary endpoint, the 6-min walk test (6MWT) which assesses distance (in meters, 6MWD) walked over 6 min, a change of 26 m is considered to be a minimal clinically relevant difference (MIREDIF) in patients with severe and very severe COPD [26, 27]. Based on a two-sample independent t-test with the given MIREDIF of 26 m, standard deviation of 44.6 m based on data published by Puhan et al. 2011 [27], power of 80% and significance level of 0.05 47 patients will be needed in each group, 94 in total. A dropout rate of 30% is assumed, resulting in 134 patients being included in the final study population.

#### Power estimations for secondary outcomes

We performed power estimations for all secondary outcomes based on the decided inclusion of 134 (67 in each group) patients, and expected standard deviation (SD) and an existing minimal clinical important difference (MCID) for each outcome (Table 2). The decided sample size makes it possible to detect clinically relevant differences in secondary outcomes for respectively; muscle strength and leg endurance, symptoms, anxiety and depression, and health-related quality of life (HRQOL) all corresponding with a power above 80% to reject the null hypothesis (type I error 5%). The outcomes for disease specific quality of life and physical activity were both have a power below 80%, and must be considered as exploratory outcomes (Table 2).

**Table 2:** Anticipated power on secondary outcomes

Variables	Instrument	Subscales	Cronbach’s alpha	Hypothesized Difference/ SD (anticipated power)
Muscle strength and endurance legs	30 s sit-to-stand test	Total numbers of repetitions	NR (not reported)	2.0/2.5 (0.99)
Symptoms	COPD Assessment Test (CAT)	Eight symptom questions (0–5 points) Total score 0–40 points	0.88	3.0/5.5 (0.88)
Disease specific quality of life	Clinical COPD Questionnaire (CCQ)	Ten items, three domain score (symptoms, functional and mental) and overall score. Items score ranges from 0 to 6	Overall score 0.91 Symptom score 0.78 Functional score 0.89 Mental score 0.80	Overall score 0.4/1.1 (0.55)
Anxiety and depression	Hospital Anxiety and Depressions Scale (HADS)	HADS-A scale (0–21) HADS-D scale (0–21)	HADS-A 0.83 HADS-D 0.82	HADS-A 1.5/2.5 (0.93) HADS-D 1.5/2.5 (0.93)
Health-Related Quality of Life	EuroQol 5-Dimension Questionnaire (EQ-5D)	EQ5D-questionnaire (mobility, self-care, usual activities, pain/discomfort, and anxiety/depression) Norm based utility score (−0.624–1.000) EQ5D-VAS (0–100 mm)	Not relevant – only one question in each dimension	EQ5D-VAS 8/16 (0.82)
Physical activity	*active*PAL™ activity monitor (PAL Technologies Ltd., Glasgow, UK)	Steps per day Minutes lying/sitting Minutes standing/walking Number of body transitions per day	NR (not reported)	Steps per day 1100/2262 (0.50)

### Study groups

#### Warm-up in both groups (CCRe and CORe)

Warm-up has duration of 5 min (in CORe group) and 10 min (in CCRe group). The aim is familiarization of movements, increasing range of motion and stimulation of joints, muscles and cardiorespiratory warm-up in accordance with recommendations from the American College of Sports Medicine [28]. The warm-up protocol is presented in Table 3.

**Table 3:** Warm-up protocol – intervention COPD online rehabilitation

Time	Exercises	Intensity	Progression
Warm-up (duration 5 min)	Sitting or standing: -Heel uprisings (uni- or bilateral), - Kneeextension - rear deltoid row - chest press movement - Vertical shoulder press’ (uni- or bilateral). Standing: -Walking on site - side to side walking - leg curl - leg swing - squats	Non-specific intensity Purpose: -increase body temperature - cardiorespiratory warm-up -muscle and tendon tissue warm-up	none

#### Comparison group - conventional COPD rehabilitation programme (CCRe)

Patients in the comparison group will receive the supervised standard COPD Rehabilitation program (CCRe) for patients with severe and very severe (stage III-IV) COPD, which follows the Danish Health Authority’s National Clinical Guideline and the Regional Guidelines [6, 29, 30]. The guidelines allow for minor variations in the duration of the program (from 10 to12 weeks) but not in the content of the program [6, 29, 30]. The rehabilitation program contains exercise and patient education. Exercise sessions last 60 min twice weekly and will be supervised by skilled physiotherapists with at least 2 years of experience with COPD rehabilitation. The content of the physical exercises is presented in Table 4. Patient education sessions of 60–90 min will take place once weekly following the exercise session. The total number of patient education sessions will vary from 10 to 12 lessons (including follow-up sessions). Topics covered in the education program are presented in Table 5.

**Table 4:** Exercise content comparison group - conventional COPD rehabilitation

Exercise type	Exercises	Intensity	Progression
Warm-up (duration 5-10 min)	Sitting or standing: -Heel uprisings (uni- or bilateral), - Kneeextension - rear deltoid row - chest press movement - Vertical shoulder press’ (uni- or bilateral). Standing: -Walking various - leg curl - leg swing - squats	Non-specific intensity Purpose: -increase body temperature - cardiorespiratory warm-up -muscle and tendon tissue Warm-up	none
Endurance training (duration 20-30 min)	-Walking or -Cycle or - Treadmill or - Circuit training or - Activity games	Borg CR-10 dyspnea 4–7 Exercises performed in intervals or continuous	Every 2nd to 4th week load adjustment individualized
Resistance training Duration 20-30 min)	Machine: -leg press -knee extension Pull down and/or chestpres (vertical) Other equipment for strength circuit training elastic band Dumbbells Weight cuff	40–80% of 1RM corresponding from 8 to 25 repetitions 2-3sets	Every 2nd to 4th week load adjustment individualized (repetition counting by supervisor)
Cool-down (duration5-10 min)	Breathing exercises Pursed lip breathe Relaxation exercises Yoga exercises	Non-specific intensity	Non-specific

Responsible health profession: Physiotherapist

Monitoring of intensity may vary, but it is expected that hospitals use either objective (pulse or Watt monitoring) or subjective (CR Borg scale for dyspnea) measurements for intensity monitoring

Resistance training will be evaluated for progression by counting their maximal repetition and estimate a new optional weight/resistance within 8–25 repetitions

Workout logs from every training session are recommended registered by the authorization law

**Table 5:** Patients educations topics control group – conventional COPD rehabilitation

Topics/themes	Communication/ learning form
• COPD and the treatment • The importance of smoking cessation • The importance of daily activity and exercise • The importance of nutrition • Medication and use of devices and inhalation technics • Early signs of exacerbation and action plan • Use of nebulizer apparatus and oxygen apparatus. Individually smoking cessation and dietician will be optional for the individual COPD patient if assessed relevant.	Topics are promoted as a combination of: • Information • Dialogue • Reflection exercises • Practical exercises • Focusing on increasing the individual’s self-competence • Networking and exchange of experience.

Responsible health profession: Respiratory nurse

#### Intervention group - COPD online rehabilitation programme (CORe)

Patients in the intervention group will receive the supervised COPD Online Rehabilitation Program (CORe), which is an intervention that has never been systematically offered in Denmark. The CORe intervention is supervised by skilled physiotherapists and respiratory nurses with at least 2 years of experience with COPD rehabilitation, and delivered via a web-cam at Bispebjerg Hospital to a group of 4–8 patients who exercise at home and communicate via a computer. Each session is 60 min, i.e. 35 min of exercise and 25 min of patient education, three times per week for the duration of 10 weeks. The exercises used in CORe exercise program were identified and selected amongst exercises used in previous exercise intervention studies on patients with severe or very COPD and involves larger muscle groups with 50/50% exercises for upper and lower extremities, respectively [4, 5, 31–36]. Volume, intensity and content specified in the training protocol is in accordance with both national and international exercise recommendations [4–6, 29, 30, 37, 38]. The exercises (Tables 6 and 7) are executed in four sets to achieve peripheral muscle fatigue and secondary dyspnea/breathlessness. Each set is carried out in a predefined period of 20 to 40 s with a maximum number of repetitions performed, i.e. 8 to 25 repetitions depending on the patients exercise capacity and motivation [28, 39], but with the aim of 12 to 20 repetitions. The pause is predefined from 40 to 20 s (Table 6). The exercise velocity is based on recommendations applying to high-repetitive exercises (> 15 repetitions) [28], i.e. moderate to high speed equaling 1–2 s for both the concentric and the eccentric movements. The exercise load is body weight supplemented by external weight using dumbbells (1 to 10 kg). The intensity is estimated to be equivalent to 40–80% of one repetition maximum (8–25 repetitions), and exercises are performed as high repetitive time-based muscle endurance training at least 80% of the exercise time corresponding to a weekly volume of 90 min (30 min × 3 sessions). In practice the training intensity is determined by using the self-rated Borg CR-10 scale (score range 0–10), and the aim is to achieve a training intensity corresponding to score 4–7 (moderate to very strong shortness of breath during the exercises).

**Table 6:** Exercise protocol intervention group COPD online rehabilitation (Chronological order)

Exercise#	Exercise name	Extremities	Uni/bilateral execution	Bodyposition	Time/volume	Exercise load
1	Sit-to-stand	Lower extremities	Bilateral	Sitting and standing	Active: 80-160 s. Rest:160-80s. Total: 240 s.	Bodyweight and dumbbells
2	Biceps curl -shoulder press	Upper extremities	Bilateral	Standing	Active: 80-160 s. Rest:160-80s. Total: 240 s.	Dumbbells
3	Step-up	Lower extremities	Bilateral	Standing	Active: 80-160 s. Rest:160-80s. Total: 240 s.	Bodyweight, dumbbells and stepbox
4	Bent Over Rowing	Upper extremities	Unilateral	Standing Upper body slightly forward bended	Active: 80-160 s. Rest:160-80s. Total: 240 s.	Dumbbells
5	Static-dynamic Squat	Lower extremities	Bilateral	Standing	Active: 80-160 s. Rest:160-80s. Total: 240 s.	Bodyweight and dumbbells
6	Front Raise Dumbbells	Upper extremities	Bilateral	Standing	Active: 80-160 s. Rest:160-80s. Total: 240 s.	Dumbbells

**Table 7:** Progression model - intervention group COPD online rehabilitation (Chronological order)

Phase	Week number	Working volume in seconds	Rest volume in seconds	Number of sets for each exercise
Familiarization	1–2	20	40	4
Progression 1	3–6	30	30	4
Progression 2	7–10	40	20	4

The first 2 weeks serve as a familiarization phase with the purpose to adapt to exercising, adjusting and optimizing load and to avoid musculoskeletal overload injuries. Thus, exercises for the lower extremities (Table 6: exercise # 1, 3, 5) are carried out without dumbbells at the first session. If a patient can perform three consecutive sets without resting during the active period external load is added at the following training session. The external load increase ranges from 2 to 4 k (total weight for two dumbbells) when progression adjustments are made. Exercises for the upper extremities (Table 6: exercise # 2, 4, 6) are carried out with the smallest weights (1 kg / pcs.) at the first exercise session. If a patient can perform three consecutive sets without resting during the active period external load is added at the following training session. The external load increase ranges from 2 to 4 k (total weight for two dumbbells) when progression adjustments are made. Progressions are assessed individually from session to session [31–34]. In addition the patients are asked to count their repetitions in each set every 6th sessions, and if the number of repetitions exceeds 25 the external load is increased at the next training session.

#### Exercise log

Each patient has an exercise log, which is completed by the supervisor who instructs the sessions online. The exercise log contains the number of completed sets, loads in kilo, customized additions and non-completed sets for each of the participants for all sessions.

#### Patient education

The education topics are disseminated as a combination of dialogue, reflection exercises and practical exercises [30, 40] (Table 8). Overall the topics are similar to those in CCRe group (Table 5), but delivered as 20 min sessions three times per week in total 30 sessions. The medical and nutrition topics are provided by a respiratory nurse. The dissemination focus is in particular on:

- Participation and dialogue to facilitate sustainable knowledge related to COPD
- Creating space for reflection and deliver opportunity for the patient’s own action plan for handling the disease
- Awareness and acceptance of patients’ different ways in understanding and acquire knowledge
- Promoting the positive aspects and opportunities in life with COPD

**Table 8:** Patients educations protocol – intervention group COPD online rehabilitation

Topic/themes	Communication/ learning form	Week	Duration	Number of sessions
Welcome and individual presentations	Information, dialogue	1	20 min	3
COPD and the treatment	Information, dialogue	2	20 min	3
Early signs of exacerbation and action plan	Information, dialogue, reflection	3	20 min	3
Medication and use of devices and inhalation technics Use of nebulizer apparatus and oxygen apparatus.	Information, dialogue, reflection, practical exercises	4	20 min	3
Physical activity and exercise	Information, dialogue, reflection	5	20 min	3
Food, importance of food in COPD	Information, dialogue, reflection, practical exercises	6	20 min	3
Smoking, cessation, substitution	Information, dialogue, reflection	7	20 min	3
Anxiety management, relaxation	Information, dialogue, reflection	8	20 min	3
Repetition		9	20 min	3
Group needs		10	20 min	3

### Statistical analysis

#### Descriptive data

Descriptive data for the intervention and comparison groups will be compared using Chi-square test or Fishers exact test for categorical variables, the Student’s t-test for normally distributed continuous variables, if the normality can’t be assumed non-parametric Mann-Whitney U test will be used instead. Descriptive variables will be presented as means, standard deviation, medians with range or frequencies with percentages depending on the distribution of the variable.

#### Analysis of primary and secondary outcome

Difference between intervention groups in primary and secondary outcomes at 10/12 week follow-up are analyzed by mixed effect models. Models will include treatment, age, gender, BMI, FEV1 and smoking status, and random effect assessor, hospital allocation and subject. Normal distribution of the model residuals is evaluated by QQ-plots, transformation of outcome may be used if distribution of residuals are not normal. All models will be fitted as listed above, in the event that convergence is not possible or other fitting issue the model structure will be reduced and reasoning for changes will be mentioned.

If all missing data is missing at random, maximum likelihood estimation in the mixed effect models can be done under assumptions of ignorability to account for missing data.

Secondary outcomes will also be analyzed mixed effect model with the same fixed and random effects as the primary outcome.

No interim analysis will be made. Statistical analyses will be carried out using R 3.2.2 (R Foundation for Statistical Computing, Vienna, Austria). *P*-values of less than 0.05 are considered statistically significant.

#### Health economic analysis

Costs related to the interventions are calculated based on the expenses associated with exercise instruction and support, the time spent by participants and relatives, transportation costs and the participants’ use of health care services. Cost-effectiveness (cost per quality-adjusted life year) is estimated from these cost calculations combined with changes in EQ-5D-5 L scores over time during the observation period. Costs related to COPD treatment and involved in the use of health care services by patients and relatives are estimated from national administrative health registries.

The Health economic analysis will be published in a separate publication and a potential business case conducted by an independent consultant company.

### Compliance

In addition to the intention-to-treat analysis, we will also perform a per-protocol analysis. The participants in both groups must have completed 70% per cent of the COPD rehabilitation program to be included in the per-protocol analysis.

### Data collection

Blinded assessors perform pre- post- and follow-up tests and collect data in CRF’s at five locations (Bispebjerg-, Hvidovre-, Gentofte-, Herlev- and Frederikssund University Hospitals) to cover the whole Capital Region. To the extent possible, the same assessor tests a participant at all test times. For practical reasons all locations have two to three assessors available. All assessors have completed a four-hour assessor course to ensure that they follow the same testing protocol, and that test procedures and recording of results are standardized. In addition, they have observed at least four live tests before being accredited to start as blinded assessors. All assessors are familiar with the physical performance tests (6MWT and 30-s Sit-To-Stand test) from clinical practice and evaluation.

### Data management

All CRF’s and paper questionnaires will be checked for errors and missing data before being entered in log-protected spreadsheet database. All entered data will be double-checked against the CRF, range checked and exported to relevant statistical software (SPSS, R, GraphPad). The principal investigator will have access to the full dataset, and co-investigators and steering committee will have access as needed for random auditing. All paper-based CRF’s and questionnaire versions will be anonymized and locked in a filing cabinet to ensure confidentiality. Data management will comply with the rules of the Danish Data Protection Agency.

### Outcomes

#### Primary outcome measure

The 6-min walk test (6MWT) will be used to assess endurance and walking capacity. The 6MWT is widely used for measurement of endurance walking capacity in patients with COPD [4, 41]. The test is performed in accordance with standardized guidelines [41]: the walking course is 20 m due to walking space shortage at some locations and to ensure same standard walking length at all five locations [42]. The patients will be instructed to walk as far as possible in 6 min; receive recommended standardized encouragement; two tests are performed to eliminate a potential learning effect and the highest value is recorded; a 30-min rest is mandatory between the first and second 6MWT.

#### Secondary outcome measures

*The 30-s sit-to-stand test (30s-STS)* will be used as an indirect assessment of lower extremity muscle strength [43, 44]. A standardized chair with a seat height of 45–47 cm is used, and the patients will be asked to stand up fully and sit down as many times as possible in 30 s with the arms across the chest. The numbers of full stands will be recorded. The score zero will be recorded if a patient is unable to rise from the chair without using the arms. Two tests will be performed to eliminate a potential learning effect, and the best result will be recorded. A 30-min rest is mandatory between first and second 30s-STS.

*COPD Assessment Test (CAT)* is a patient completed 8-item questionnaire that assesses the impact of COPD on self-reported health status and symptoms [45]. Each item scores from 0 to 5 points (0 indicating no impact or symptoms, 5 worst possible impact or symptoms) summing up to a total CAT score range of 0–40 points. CAT is a validated tool with a Cronbach’s α of 0.88 and found responsive to change in self-reported health status and symptoms after pulmonary rehabilitation [45–47]. A minimal clinical important difference (MCID) of 2–3 points is suggested [47, 48].

*Clinical COPD Questionnaire (CCQ)* measures self-reported quality of life [49]. CCQ consist of 10-itmes with an overall score and 3-domain scores: Symptoms (4-Items), Functional state (4-Items) and Mental state (2-Items) [49]. Overall- and domain scores ranges from 0 to 6 (0 = no impairment). The CCQ is a validated tool with a Cronbach’s α of 0.91 for the total score and 0.78, 0.89, 0.80 respectively for the symptoms, functional and mental scores and has a interclass correlation coefficient of 0.94 [49]. A MCID of 0.4 point is suggested [50, 51].

*Hospital Anxiety and Depressions Scale (HADS)* is a 14-item questionnaire that assesses anxiety and depression level in medically ill persons [52]. The scale offers two sub scores HADS anxiety (HADS-A) and HADS depression (HADS-D), and consists of seven questions to assess anxiety and seven questions to assess depression. Each question scores from 0 to 3 (0 = no symptoms). HADS is a validated tool with Cronbach’s α of 0.83 (HADS-A) and 0.82 (HADS-D) [52]. Scores of 0–7 from each of the two subs scales are considered normal and scores of 8–10 suggest a risk of anxiety and/or depression disorder. Scores of 11 and above suggest the probable presence of anxiety and/or depression disorder [52]. A MCID of 1.5 point in each scale is suggested based on both anchor and distribution-based methods [53].

*EuroQol 5-Dimension Questionnaire (EQ-5D)*, is generic self-reported global measure for health-related quality of life [54]. EQ-5D compromises a 5-dimension (mobility, self-care, usual activities, pain/discomfort, and anxiety/depression) questionnaire and a 20 cm visual analog scale (EQ-VAS) ranging from zero (worst imaginable health) to 100 (best imaginable health) [54]. A MCID of 8-points in EQ-VAS is suggested in persons with COPD, while no MCID is established in the 5-dimension questionnaire in persons with COPD [55].

*24-h physical activity* is measured with an *active*PAL ™ triaxial accelerometer (PAL Technologies Ltd., Glascow, UK). The patients will be asked to wear an *active*PAL™ on the thigh 24 h per day for 5 days prior to randomization; 5 days during the intervention period (after 5–7 weeks); 5 days after completion of intervention period; 5 days 3 months after intervention completion; and 5 days 12 months after completion of the intervention. The 24-h physical activity level is an exploratory outcome in this study. Due to limited staff resources and geographical transportation issues activity level will only be measured in the first 68 patients (approximately 50% of the population) who live within a radius of 25 km from Bispebjerg University hospital. The *active*PAL™ accelerometer is attached on the front of the thigh and measures time spent lying/sitting (thigh in horizontal position), and time spent standing and walking (thigh in a vertical position), the number of steps taken, cadence, and the number of sit-to-stand and stand-to-sit transitions. The *active*PAL™ is a valid and reliable measure of posture and transitions in mobility limited older adults and adults with severe and very severe COPD [56–58]. A MCID of 600–1100 steps per day is suggested in persons with COPD, depending on which distribution based method you rely on [59]. However *active*PAL™ underestimates step rate at slow walking speeds compared to observed step counts, while use of walking aid such as rollator and crutches do not differ from observed step rate counts [58]. Walking speed between 2.4–5.6 km/h is preferable to get valid data on time spent walking [56, 60] and consequently walking could potentially be categorized as standing in those walking slower 2.4 km/h [56, 60]. For this reason we will dichotomize data into time spent sedentary (lying/sitting) and upright (standing/walking).

### Reliability calculations

Intra- and inter tester reliability will be calculated on physical outcome measures (6MWT and 30s-STS) in fifty consecutively recruited patients. The retest is completed seven to 10 days after baseline assessment and prior to intervention start. The reliability in the patient reported questionnaires (CAT, CCQ, HADS, EQ-5D) will be calculated in the same sample of fifty patients.

The reliability for the physical outcome measures and patient reported outcomes will be published in separate articles.

### Other variable and outcomes

Other variables and outcomes will be registered as mandatory in the hospital registry system and used as descriptive as well as explanatory variables for the primary and secondary outcomes (Table 1).

### Demographic, descriptive variables

Age, gender, height, weight, body mass index, marital status, education, smoking status, years with COPD, Charlson morbidity index and lung medications are registered at baseline assessment.

The lung spirometry is conducted at the respiratory department of the referral hospital by a lung physician or respiratory nurse before baseline and 12-month follow-up assessments. All hospitals use clinically approved spirometry equipment while manufacture trademark varies between hospitals in the Capital Region (Table 1).

### Publication process

HH are obligated to ensure that the results of the study are published in due time after completion of the study.

### Changes to initial plan

In the initial registration of the study, we planned to do the outcome measure pre-, post- and 3 month follow-up on the performance- and self- reported outcome measures (Fig. 1 and Table 1), while 6-month, 12-month follow-up should be based on register data defined as number of hospital admission related to COPD, number of hospitals admission days related to COPD, number of outpatient visits related to COPD and mortality. After reconsideration, the steering committee decided to prepare an additional protocol and apply the ethical committee for permission to perform 12 months of follow up on performance- and self- reported outcome measures. All patients already included will be asked retrospectively to enter and participate in a 12 month follow up test. Prospectively participation in the 12 months of follow up is not mandatory to be included in the CORe trial. A new informed consent form is filled if the participant accepts “extra” participation in the 12 months of follow up.

### Adverse event reporting

Adverse events are recorded in the CRF. The protocol distinguishes between adverse events that may be directly attributable to the study interventions and the monitoring of adverse events not attributable to the study. Serious adverse events are reported within 24 h to the principal investigator. The steering committee consisting of a pulmonologist, respiratory nurse and clinical physiotherapist surveys the study and evaluates serious adverse events.

## Discussion

To our knowledge there are currently no RCT studies published comparing supervised outpatient COPD rehabilitation in groups with supervised COPD tele-rehabilitation in groups [13–21]. McCarthy et al. 2015 confirmed that COPD rehabilitation indeed is beneficial in clinical trials [2]. However in the real world COPD rehabilitation, we know that compliance amongst the patients with COPD is low and potentially insufficient due to frequent exacerbation, hospitalization, transportation dependency, uniform offered outpatient COPD rehabilitation in hospitals, municipalities and other outpatient settings. This study provides a detailed description of a simple, fully supervised COPD Online rehabilitation program, which aims to provide high frequent attendance with a minimal time consuming exercise and educational approach. The choice of exercises, equipment, frequency and duration was chosen in order to investigate whether a few specific but simple exercises, can improve endurance, strength, physical activity, symptoms related to COPD and HRQOL.

This approach was chosen to facilitate implementation of a minimally equipped home based online supervised COPD rehabilitation program, which might be more feasible and thereby more efficient than conventional COPD rehabilitation in patients with severe and very severe COPD. A problem in previous exercise studies is the lack of standardization which makes reproducibility difficult. The comprehensive standardization of exercise and progression protocol in this trial facilitates the reproducibility of this trial.

The results from this study will most likely provide important knowledge regarding treatment of Patients with COPD who are unable or non-compliant to conventional offered COPD rehabilitation settings. The study will also contribute to the existing knowledge regarding reasons for accepting and declining participation in CCRe and CORe, characteristics of completers and non-completers, and the long term effects of the two rehabilitation approaches. The CORe-Trial is designed from high quality criteria in a non-pharmacological clinical trial, with a multicenter design which reduces risk of selection bias. Assessors and biostatistician are blinded to intervention, which should reduce detection and interpretation bias. The research group will be blinded when interpreting data and writing the conclusion.

### Trajectory

Inclusion was initiated 18th of March 2016 and will continue until 31st of December 2017.

## Abbreviations

30sec-STS: 30 seconds sit-to-stand test
6MWD: Six minute walking distance
6MWT: Six minute walking test
BMI: Body mass index
CAT: COPD assessment test
CCQ: Clinical COPD questionnaire
CCRe: COPD conventional rehabilitation
CI: Confidence interval
CONSORT: Consolidated standards of reporting trials
COPD: Chronic obstructive pulmonary disease
CORe: COPD online rehabilitation
CRF: Case report form
EQ-5D: EuroQol 5-dimension questionnaire
FEV1: Forced expiratory volume at one second
HADS: Hospital anxiety and depressions scale
HRQOl: Health-related quality of life
MCID: Minimal clinical important difference
MIREDIF: Minimal clinically relevant difference
MRC: Medical Research Council
PA: Physical activity
RCT: Randomized controlled trial
SD: Standard deviation
SPIRIT: Standard protocol items recommendations for interventional trials
TIDieR: Template for interventions description and replication
